# The Impact of Chronic Kidney Disease on Mid-Term Outcomes after Revascularisation of Peripheral Arterial Occlusive Disease: Results from a Prospective Cohort Study

**DOI:** 10.3390/jcm11164750

**Published:** 2022-08-14

**Authors:** Artur Kotov, Deven A. Blasche, Frederik Peters, Philip Pospiech, Ulrich Rother, Konstantinos Stavroulakis, Jürgen Remig, Christian Schmidt-Lauber, Thomas Zeller, Hartmut Görtz, Jörg Teßarek, Christian-Alexander Behrendt

**Affiliations:** 1 Research Group GermanVasc, University Medical Centre Hamburg-Eppendorf, 20246 Hamburg, Germany; 2 Department of Vascular Surgery, University Medical Centre Erlangen, 91054 Erlangen, Germany; 3 Commission on Peripheral Artery Disease and Diabetic Foot Syndrome, German Society for Vascular Surgery and Vascular Medicine, 10115 Berlin, Germany; 4 Department of Vascular Surgery, Ludwig Maximilians University Hospital, 80539 Munich, Germany; 5 Bonn Community Hospital, Haus St. Petrus, 53113 Bonn, Germany; 6 III. Department of Medicine, University Medical Centre Hamburg-Eppendorf, 20246 Hamburg, Germany; 7 Clinic for Cardiology and Angiology II, University Heart Center Freiburg-Bad Krozingen, 79189 Bad Krozingen, Germany; 8 St. Boniface Hospital Lingen, 49808 Lingen, Germany; 9 Brandenburg Medical School Theodor Fontane, 16816 Neuruppin, Germany

**Keywords:** chronic kidney disease, end-stage kidney disease, lower extremity artery disease, intermittent claudication, chronic limb-threatening ischaemia, open and endovascular revascularisation

## Abstract

Objective: The current study aimed to determine the relationship between chronic kidney disease (CKD) and major 12-month outcomes for patients with in-hospital treatment for symptomatic peripheral arterial occlusive disease (PAOD). Methods: An analysis of the prospective longitudinal multicentric cohort study with 12-month follow-up was conducted including patients who underwent endovascular or open surgery for symptomatic PAOD at 35 German vascular centres (initial study protocol: NCT03098290). Severity of CKD was grouped into four stages combining information about the estimated glomerular filtration rate (eGFR) at baseline and dialysis dependency. Outcomes included overall mortality as well as the two composite endpoints of amputation or death, and of major cardiovascular events (MACE). 12-month incidences and adjusted hazard ratios were estimated using the Kaplan–Meier function and Cox proportional hazard models. Results: A total of 4354 patients (32% female, 69 years mean age, 68% intermittent claudication, 69% percutaneous endovascular revascularisation) were included and followed for 244 days in median. Thereof, 22% had any CKD and 5% had end stage kidney disease (ESKD) at baseline. The 12-month overall mortality rate was 3.6% (95% CI 2.3–4.9) with 96 events in the entire cohort: 147 were amputated or died (5.3%, 95% CI 5.2–5.3), and 277 had a MACE (9.5%, 95% CI 9.4–9.5). When compared with patients without kidney disease, ESKD was significantly associated with overall mortality (HR 1.9; 95% CI 1.1–3.5), amputation or death (HR 2.4; 95% CI 1.4–4.1), and MACE (HR 2.0; 95% CI 1.3–3.2). Conclusions: In the current study on mid-term outcomes after invasive revascularisation for symptomatic PAOD, one out of five patients suffered from any CKD while those few with ESKD had twice the odds of death, of amputation or death, and of major adverse cardiovascular events after twelve months. These results emphasise that concomitant CKD and its impact on outcomes should be considered by severity while mild and moderate grades should not lead to ineffectual treatment strategies.

## 1. Introduction

The management of lower extremity peripheral arterial occlusive disease (PAOD) and its consequences is a global public health challenge [1,2,3]. In 2015, approximately 237 million people were affected by this common disease [1,2]. Moreover, the impact of diabetes on the development and long-term course of cardiovascular disease significantly expands the burden of this growing target population. Chronic kidney disease (CKD) counts as an important risk factor for cardiovascular disease and vice versa [4,5,6,7,8,9]. Several studies have shown that CKD was frequent amongst patients with PAOD, and that kidney disease was further associated with worse outcomes. Interestingly, evidence on the exact pathways and how these both concomitant and overlapping diseases are linked with each other remains sparse to date [6,10,11].

In patients with PAOD and diabetes, arteries below the knee are more frequently affected and the lesion characteristics often comprise multilevel stenosis and occlusions. Furthermore, diabetes was found to be a significant risk factor for the development of restenosis which may lead to higher reintervention rates [12,13,14]. An association between chronic limb-threatening ischaemia and frailty was previously reported that further contributes to the complex interaction between PAOD, diabetes, and CKD [15].

The link between PAOD and CKD was underlined recently in a contemporary cohort of 5608 patients who underwent invasive revascularisation for symptomatic PAOD in Germany; 22% had CKD and 2.5% were on chronic haemodialysis at baseline [16]. In another large cohort study from the United States, 27% of 39,441 invasively revascularised patients with symptomatic PAOD had concomitant CKD. Thereby, CKD was associated with an increased risk for major adverse cardiovascular events (MACE), lower extremity amputation, bleeding, and higher rates of hospital readmission [11].

To date, the available evidence is mainly based on retrospective observational studies and there is a paucity of mid-term outcomes [5,7,11,17,18]. Thus, evidence-based recommendations to guide the therapy of patients with CKD are still lacking while valid practice guidelines also do not contain strong recommendations concerning screening strategies in cohorts at risk [4,7,10,19,20,21,22]. These gaps in knowledge and the need for further studies on the combination of PAOD and CKD was highlighted just recently in a conference conclusion of the Kidney Disease: Improving Global Outcomes (KDIGO).

The current study aimed to determine the independent impact of both CKD and end stage kidney disease (ESKD) on mid-term outcomes after revascularisation in patients with symptomatic PAOD using validated clinical data from the prospective GermanVasc cohort study (NCT03098290) [16,23].

## 2. Materials and Methods

This was a secondary analysis of a prospective longitudinal multicentre cohort study. The rationale and design of the GermanVasc registry study were already published and have been registered at Clinicaltrials.gov (NCT03098290) and the German Registry of Clinical Trials (DRKS00014649) [16,23]. The study was approved by the ethical committee at the medical association in Hamburg, Germany (PV5691) as well as 18 additional ethical committees in Germany. The European Union (EU) General Data Protection Regulation (GDPR) compliant GermanVasc registry platform was developed to follow the principles of privacy by design while collecting the personal and medical data relevant for the current study [23,24,25]. Results were reported using the STrengthening the Reporting of OBservational studies in Epidemiology (STROBE) statement [26].

### 2.1. Inclusion and Exclusion Criteria

During the study period between 1 May 2018 and 31 March 2021, patients invasively revascularised for symptomatic PAOD, denoted as index stay, were enrolled consecutively by 35 participating legally endorsed multidisciplinary German hospitals, provided written informed consent and age ≥18 years.

Exclusion criteria were lack of informed consent and acute limb ischaemia without previous history of symptomatic PAOD. Target follow-up duration was one year, while follow-ups were scheduled at 3, 6 and 12 months with a tolerance of ±21 days until 31 March 2021. The data collection underwent comprehensive completeness and plausibility checks as well as external site visit monitoring by a professional clinical trial organisation (CTC North GmbH & Co. KG, Hamburg, Germany) for ensuring high internal and external validity. According to the modified Rutherford classification, patients selected for invasive revascularisation with mild, moderate, and severe claudication were pooled as intermittent claudication (IC), while those with ischaemic rest pain and wound healing disorders were pooled as chronic-limb threatening ischemia (CLTI).

### 2.2. Study Variables

All study variables were put together and defined according to three modified Delphi studies with international registry experts [27,28,29].

### 2.3. Definition of CKD

The estimated GFR (eGFR) was used involving the serum creatinine in mg/dl and a standardised body surface of 1.73 m^2^ to distinguish between grade I to V (using the Kidney Disease Improving Global Outcomes, KDIGO classification). The severity of CKD was defined as no CKD (GFR: ≥60 mL/min/1.73 m^2^), mild CKD (GFR: 30–59 mL/min/1.73 m^2^), severe CKD (GFR < 30 mL/min/1.73 m^2^), and ESKD (dialysis dependency) [30].

### 2.4. Other Variables

Symptomatic PAOD was assessed by using the ankle-brachial index (ABI) measurements at both limbs (<0.9) and subsequently split for each limb by IC and CLTI. We used the following variables available in the GermanVasc study database: patient sex (female, male), age in years (continuous), CLTI vs. IC, index treatment (endovascular revascularisation, bypass surgery, endarterectomy, hybrid revascularisation), the American Society of Anesthesiologists (ASA) Classification (class I or II, class III or worse), employment status (retired, working, unemployed or on sick leave), smoking status (never, former or current smoker), obesity (following the World Health Organization definition at a body mass index of 30 kg/m^2^ or higher), self-rated health status (good, bad), ambulation impairment (no, yes if use of prosthesis, walking aid or wheelchair), prior coronary revascularisation (none, any), prior peripheral revascularisation (none, endovascular intervention or open surgery only, both endovascular intervention and open surgery), diabetes (no, yes without insulin use, yes with insulin use), history of congestive heart failure (no, yes), lipid-lowering drugs at admission (no, yes), antiplatelet drugs at admission (no, yes).

Patients with missing information regarding Rutherford classification, sex (*n* = 148), age (*n* = 29), urgency of admission (*n* = 62), obesity (*n* = 162), lipid-lowering drugs (*n* = 106) and antiplatelet drugs (*n* = 23) were excluded. For the remaining variables, missing data were assigned to a separate category, respectively.

### 2.5. Study Endpoints

Study outcomes were (a) overall death (b) major target-limb amputations and/or death, (c) major adverse cardiovascular events.

All events were recorded at index stay, including unplanned amputations and in- hospital deaths, and up until 365 days after discharge. After discharge, death events were measured at the date of occurrence and all other events at the date of the follow-up interview. For each patient, the revascularised limb at index stay was noted (left, right or both) for identifying target-limb related amputation endpoints.

If patients did not experience an event of interest within 365 days, right censoring was applied at the date lost to follow-up or at date of discharge from index stay if no follow-up information was available.

### 2.6. Statistical Analysis

Descriptive patient characteristics were expressed as means and standard deviation (SD) and proportions if not stated otherwise. The assumption of normality of the data was evaluated by visual inspection of the corresponding histograms and diagrams. Exact post-discharge event times for all events other than death were imputed using a parametric Cox proportional hazards for interval censored data with Weibull baseline distribution with age as explanatory variable. The 12-months incidence and 95% confidence interval (CI) for each endpoint was estimated from a Kaplan–Meier function with cluster bootstrapping with one thousand replications accounting for correlation of patients within medical centres. Unadjusted and adjusted risk differentials among the four study groups were expressed as hazard ratios (HR) extracted from Cox proportional hazard models. The proportionality assumption was assessed graphically. For adjusting the models, the sixteen explanatory variables as defined above were employed. Missing values were imputed using multivariate imputation by chained equations separately for five copies of the data with twenty iterations. Estimates were averaged and standard errors adjusted using Rubin’s rules. There were no adjustments made for multiple comparisons. All analyses were performed with software R version 4.1.1 (The R Foundation for Statistical Computing, Vienna, Austria).

## 3. Results

Table 1 summarises the baseline characteristics of the entire cohort stratified by IC vs. CLTI. A total of 4354 patients (31.6% female; median age 69 years) underwent revascularisation for symptomatic PAOD (68.4% with IC, 68.6% percutaneous endovascular revascularisation). The median follow-up time was 244 days.

Of these, 935 (21.5%) had CKD and 102 (2.3%) had ESKD. Regarding patients with CLTI, 30.2% had CKD and 5% had ESKD. In contrast, 17.4% of all patients with IC had CKD and 1.1% ESKD. In 4.7% of all patients, both lower limbs were invasively treated and 41.7% reported a prior peripheral revascularisation.

### 3.1. Mid-Term Incidence of Events by Severity of Chronic Kidney Disease

The 12-month incidences of overall mortality, amputation or death, and major cardiovascular events for all patients by severity of CKD are reported in Table 2.

After twelve months, the all-cause mortality for the entire cohort was 3.61% (95% CI 2.33–4.89). There was a significant increase of mortality rates with CKD severity, being highest in patients with ESKD (16.49%, 95% CI 5.62–27.36). However, compared with the 3099 patients without CKD (2.45%, 95% CI 1.36–3.53), there was a trend towards higher overall mortality even in patients with moderate (5.35%, 95% CI 3.13–7.57) and severe CKD (8.11%, 95% CI 2.14–14.07).

The 12-month incidence of the composite of amputation and death in the entire cohort was 5.27% (95% CI 5.23–5.32). The highest rates were observed in patients with ESKD (29.31%, 95% CI 28.73–29.88).

The 12-month incidence of major adverse cardiovascular events in the entire cohort was 9.45% (95% CI 9.4–9.49). The highest incidence was observed in patients with ESKD (35.45%, 95% CI 34.71–36.2).

### 3.2. Association between Chronic Kidney Disease and Outcomes in Unadjusted vs. Adjusted Analyses

Both the unadjusted and adjusted hazards are presented in Table 3. During the follow-up, 96 deaths occurred, 147 patients exhibited the composite of amputation or death, and 277 patients had a major adverse cardiovascular event.

In the unadjusted models, any CKD (moderate, severe, ESKD) was statistically significantly associated with worse outcomes.

In the adjusted models, both severe CKD (HR 1.66, 95% CI 1.06–2.60) and ESKD (HR 2.04, 95% CI 1.30–3.21) were associated with higher rates of MACE when compared with no kidney disease (reference) while only ESKD was also associated with overall mortality (HR 1.93, 95% CI 1.07–3.49) and amputation or death (HR 2.41, 95% CI 1.41–4.13) (Table 3).

## 4. Discussion

The current large observational study used validated data from a prospective multicentric cohort study to demonstrate that mid-term outcomes after invasive revascularisation of patients with both symptomatic PAOD and concomitant ESKD are quite poor.

In appropriately adjusted analyses, patients with ESKD had higher overall mortality rates, exhibited the composite of amputations or death more often, and had higher MACE rates than their counterparts without kidney disease. The latter cardiovascular endpoint was also more frequent in patients with severe CKD. While it has been known for some time that CKD is an important cardiovascular risk factor, the current study added the first evidence on independent impact of impaired kidney function by specific eGFR levels. The applied models were adjusted for the most commonly accepted confounders and generated robust results while several previous studies failed to adjust for the confounding by diabetes and other comorbidities.

Except for a few observational studies, there has been a paucity of data on the impact of both CKD and ESKD on outcomes after revascularisation of symptomatic PAOD [5,7,11,17,18,21]. While several previous studies used administrative registries and reimbursement codes with large numbers to determine the complex interactions of kidney disease with cardiovascular disease, there is evidence that billing codes are not a completely accurate way to identify advanced kidney disease stages [31,32]. To overcome this vital limitation, the current study used externally validated eGFR values to identify the variable of interest with good granularity. Thereby, it was also possible to apply the most recent formula to calculate the appropriate KDIGO grades.

Patients with ESKD were previously considered a high risk-group by clinical guidelines while these patients were often excluded from trials and studies [7,33]. Even if patients with CKD were included, studies often fall short in considering additional risk factors that are associated with CKD such as diabetes. This may have driven the fact that recent guidelines do not contain strong recommendations on the management of these patients [4,19].

The present study’s results confirmed recent studies on CKD and ESKD involving the same target population. Interestingly, in spite of the difference in the temporal aspect of outcomes, Smilowitz et al., also described a severity dependent association between CKD and adverse short-term outcomes after revascularisation. Notably, patients with ESKD had the highest risk regarding in-hospital death, amputation and bleeding [11]. In a recent systemic review and meta-analysis of long-term outcomes after lower extremity revascularisation in patients with CKD and ESKD, Anantha-Narayanan et al., also described a higher risk for all-cause mortality and major amputation in patients with CKD and ESKD regardless of the type of revascularisation [7]. Strikingly, the worst long-term outcomes were also described in patients with ESKD. However, no precise analyses of the severity of CKD and adverse outcomes were performed to date. There was only a distinction between CKD regardless of other severity levels, ESKD and normal kidney function. CKD-specific risk factors were associated with higher severity of CKD as well as higher rates of mortality and morbidity [18]. The overlap of CKD-specific and cardiovascular risk factors and the described burden of the risk factors in all patients of the present study might be a reason for the aforementioned propensity of worse outcomes in patients with CKD or ESKD. Thereby, it appears challenging to distinguish between overlapping risks on the one hand and the well-known fact that severe CKD leads to vascular damage and impaired micro perfusion on the other hand. It appears also likely that the treatment practice and decision making may be influenced by the occurrence of severe CKD or ESKD. Hence, a less rigorous revascularisation of both coronary and peripheral arteries in patients with CKD may also contribute to the higher rate of adverse outcomes.

Furthermore, the absence of optimal medical management in all patients might also be a reason for worse outcomes which might also indicate the persistent unawareness of the role of antithrombotics and lipid-lowering drugs.

The underlying study’s results filled in the current gap in terms of the lack in data on outcomes after invasive therapy of symptomatic PAOD in patients with concomitant CKD or ESKD. As illustrated here, the association between kidney disease and adverse outcomes after revascularisation in patients with symptomatic PAOD raises the question whether such patients, especially the high-risk patients with ESKD, would benefit by a multidisciplinary approach in both conservative and invasive therapy, i.e., a collaboration between vascular surgery, radiology, angiology, cardiology, and nephrology. Multidisciplinary “foot teams” following the role model of previously established “heart teams” may be an appropriate way to follow the recommendations for a stepwise care system. Unfortunately, there is striking data suggesting that multidisciplinary team discussions are still the Achilles heel of PAOD treatment [34]. To provide more data for the establishment of evidence-based therapy, future studies should avoid an exclusion of patients with concomitant CKD or ESKD as there was a severity dependent increase of the incidence of adverse outcomes. Furthermore, randomised trials with subgroup analyses on outcomes after revascularisation in patients with CKD and ERSD are warranted.

Both CKD and dialysis dependency were recently identified by machine learning methods using a large volume of administrative data to be strong predictors for worse amputation-free survival and major bleeding events after inpatient treatment (https://score.germanvasc.de (accessed on 1 August 2022)) [35,36]. Moreover, the ongoing controversy on the relationship between sodium glucose cotransporter 2 (SGLT2) inhibitors and lower extremity amputations emphasized that there may be a complex interaction between PAOD, kidney disease, and diabetes [37,38]. These interesting findings along with the current study results may illuminate the possible scope for improvement of overall unsatisfactory outcomes.

Aside from the underlying prospective longitudinal multicentre cohort study’s strengths, there were also limitations. The patient selection and choice of therapy approach was at the participating physicians’ discretion. This prospective observational study used routinely collected data and the non-random assignment made it impossible to derive a causal relationship between treatment strategy and outcomes. Thus, the present study does not give an answer to the pending question of whether an endovascular or an open revascularisation is more beneficial to patients with CKD or ESKD. In additiono, proteinuria as a separate risk kidney-derived factor for cardiovascular diseases was not assessed in this study.

## 5. Conclusions

In the current study on mid-term outcomes after invasive revascularisation for symptomatic PAOD, one out of five patients suffered from any chronic kidney disease while those few with end stage kidney disease had twice the odds of death, of amputation or death, and of major adverse cardiovascular events after twelve months. These results emphasise that concomitant chronic kidney disease and its impact on outcomes should be considered by severity while moderate grades should not lead to ineffectual treatment strategies.

## Figures and Tables

**Table 1:** Baseline characteristics of the full cohort stratified by severity of peripheral arterial occlusive disease. BMI: Body-mass-index, IQR: Interquartile range, ASA: American Society of Anesthesiologists.

	Full Cohort (*n* = 4354)	Intermittent Claudication (*n* = 2967)	Chronic Limb-Threatening Ischaemia (*n*= 1387)
Follow-up time in days, median [IQR]	224.00 [0.00, 365.00]	270.00 [47.50, 366.00]	188.00 [0.00, 362.00]
Demographics/general			
Male sex	2979 (68.4)	2030 (68.4)	949 (68.4)
Age, median [IQR]	69.00 [62.00, 77.00]	68.00 [61.00, 76.00]	72.00 [64.00, 79.00]
Index stay			
Endovascular revascularisation	2988 (68.6)	2173 (73.2)	815 (58.8)
Bypass surgery or endarterectomy	1081 (24.8)	633 (21.3)	448 (32.3)
Hybrid revascularisation	285 (6.5)	161 (5.4)	124 (8.9)
Both limbs treated simultaneously	205 (4.7)	168 (5.7)	37 (2.7)
ASA class I or II	1609 (37.0)	1252 (42.2)	357 (25.7)
ASA class III or worse	1562 (35.9)	877 (29.6)	685 (49.4)
Kidney disease			
End stage kidney disease and dialysis dependent	102 (2.3)	32 (1.1)	70 (5.0)
Chronic kidney disease	935 (21.5)	516 (17.4)	419 (30.2)
Serum creatinine in mg/dL (median [IQR])	0.95 [0.80, 1.20]	0.93 [0.80, 1.10]	1.00 [0.80, 1.40]
Comorbidities			
Obesity (BMI ≥30 kg/m²)	920 (21.1)	612 (20.6)	308 (22.2)
Diabetes			
None	2762 (63.4)	2046 (69.0)	716 (51.6)
Yes without insulin use	871 (20.0)	567 (19.1)	304 (21.9)
Yes with insulin use	721 (16.6)	354 (11.9)	367 (26.5)
Prior coronary revascularisation	682 (15.7)	427 (14.4)	255 (18.4)
Prior peripheral revascularisation			
No prior peripheral revascularisation	2145 (49.3)	1508 (50.8)	637 (45.9)
Either endovascular or open-surgery	1665 (38.2)	1144 (38.6)	521 (37.6)
Both endovascular and open surgery	544 (12.5)	315 (10.6)	229 (16.5)
Chronic heart failure	758 (17.4)	410 (13.8)	348 (25.1)
Self-rated health = bad (%)	1536 (35.3)	904 (30.5)	632 (45.6)
Ambulatory impairment	749 (17.2)	230 (7.8)	519 (37.4)
Smoking: Never smoker	3565 (35.6)	1718 (36.1)	1847 (35.2)
Smoking: Former smoker	4406 (44.1)	2042 (43.0)	2364 (45.1)
Smoking: Current smoker	1978 (19.8)	955 (20.1)	1023 (19.5)
Employment status: Retired	2002 (46.0)	1276 (43.0)	726 (52.3)
Employment status: Working	697 (16.0)	565 (19.0)	132 (9.5)
Employment status: Unemployed	468 (10.7)	323 (10.9)	145 (10.5)
Lipid-lowering drugs	3061 (70.3)	2142 (72.2)	919 (66.3)
Antiplatelet therapy	3778 (86.8)	2634 (88.8)	1144 (82.5)

**Table 2:** 12-month incidence of overall mortality, amputation or death and major cardiovascular events for the full cohort and stratified by severity of CKD. Estimates were extracted from five imputed datasets with ten iterations each and combined using Rubin’s rules. CKD: Chronic kidney disease, CI: Confidence interval.

	Overall Mortality (95% CI)	Amputation or Death (95% CI)	Major Adverse Cardiovascular Events (95% CI)
Full cohort (*n* = 4354)	3.61 (2.33–4.89)	5.27 (5.23–5.32)	9.45 (9.4–9.49)
CKD, none (*n* = 3099)	2.45 (1.36–3.53)	3.81 (3.64–3.99)	7.44 (7.26–7.62)
CKD, moderate (*n* = 1007)	5.35 (3.13–7.57)	6.7 (6.07–7.32)	11.71 (10.83–12.59)
CKD, severe (*n* = 145)	8.11 (2.14–14.07)	11.98 (10.88–13.09)	22.53 (19.61–25.45)
End stage kidney disease (*n* = 102)	16.49 (5.62–27.36)	29.31 (28.73–29.88)	35.45 (34.71–36.2)

**Table 3:** Association between severity of CKD and 12-month overall mortality, amputation or death, and major adverse cardiovascular events. Estimates were extracted from five imputed datasets with ten iterations each and combined using Rubin’s rules. CKD: Chronic kidney disease, CI: Confidence interval. * Models adjusted for age, Fontaine stage, sex, procedure type, smoking status, ambulatory impairment, obesity, self-rated health, employment status, ASA (American Society of Anesthesiologists) classification, prior coronary revascularisation, prior peripheral revascularisation, diabetes, chronic heart failure, lipid-lowering drugs, antiplatelets with cluster-robust standard errors for medical centre. # denotes statistical significance.

	Overall Mortality (95% CI)	Amputation or Death (95% CI)	Major Adverse Cardiovascular Events (95% CI)
Events	96	147	277
Unadjusted models			
CKD, none (*n* = 3099)	Reference	Reference	Reference
CKD, moderate (*n* = 1007)	2.23 (1.32–3.77) #	1.80 (1.16–2.80) #	1.64 (1.31–2.05) #
CKD, severe (*n* = 145)	3.91 (2.21–6.92) #	3.83 (2.14–6.83) #	3.18 (2.14–4.71) #
End stage kidney disease (*n* = 102)	6.65 (3.54–12.53) #	8.34 (5.26–13.22) #	4.64 (3.13–6.88) #
Adjusted models *			
CKD, none (*n* = 3099)	Reference	Reference	Reference
CKD, moderate (*n* = 1007)	1.05 (0.54–2.03)	1.04 (0.61–1.79)	1.14 (0.85–1.52)
CKD, severe (*n* = 145)	1.12 (0.57–2.23)	1.41 (0.85–2.34)	1.66 (1.06–2.6) #
End stage kidney disease (*n* = 102)	1.93 (1.07–3.49) #	2.41 (1.41–4.13) #	2.04 (1.30–3.21) #

## Data Availability

The study data is available from the corresponding author on reasonable request.
